# The Roles of Host Noncoding RNAs in *Mycobacterium tuberculosis* Infection

**DOI:** 10.3389/fimmu.2021.664787

**Published:** 2021-05-19

**Authors:** Li Wei, Kai Liu, Qingzhi Jia, Hui Zhang, Qingli Bie, Bin Zhang

**Affiliations:** ^1^ Department of Laboratory Medicine, Affiliated Hospital of Jining Medical University, Jining Medical University, Jining, China; ^2^ Nursing Department, Affiliated Hospital of Jining Medical University, Jining Medical University, Jining, China; ^3^ Institute of Immunology and Molecular Medicine, Jining Medical University, Jining, China; ^4^ Institute of Forensic Medicine and Laboratory Medicine, Jining Medical University, Jining, China

**Keywords:** *Mycobacterium tuberculosis* (*M. tuberculosis*), miRNA, piRNA, circRNA, lncRNA, immune response

## Abstract

Tuberculosis remains a major health problem. *Mycobacterium tuberculosis*, the causative agent of tuberculosis, can replicate and persist in host cells. Noncoding RNAs (ncRNAs) widely participate in various biological processes, including *Mycobacterium tuberculosis* infection, and play critical roles in gene regulation. In this review, we summarize the latest reports on ncRNAs (microRNAs, piRNAs, circRNAs and lncRNAs) that regulate the host response against *Mycobacterium tuberculosis* infection. In the context of host-*Mycobacterium tuberculosis* interactions, a broad and in-depth understanding of host ncRNA regulatory mechanisms may lead to potential clinical prospects for tuberculosis diagnosis and the development of new anti-tuberculosis therapies.

## Introduction

Tuberculosis (TB), which is caused by the intracellular pathogen *Mycobacterium tuberculosis (M. tuberculosis*), remains the leading cause of death from a single infectious agent (ranking higher than HIV/AIDS). According to the WHO global TB report, there were 10 million new cases of infection and 1.5 million TB-related deaths in 2018 (1). Drug-resistant TB has become a challenge for treating TB infection. In 2018, there were approximately 5 million new rifampicin-resistant TB cases (78% of which were multidrug-resistant (MDR)-TB) (1). The prevalence of TB is far more extensive than previously estimated.

The development of modern antibiotics and vaccines has helped humans overcome many infectious diseases, but TB has still not been eradicated. The main reasons are that *M. tuberculosis* rapidly exhibits drug-resistant mutations under the pressure of antibiotics and that the development of new TB vaccines and effective anti-tuberculosis drugs is prolonged (2, 3). Another reason is that during the evolutionary processes involved in coexisting with the host for thousands of years, *M. tuberculosis* has evolved with a set of almost perfect immune escape mechanisms that enable *M. tuberculosis* to skillfully avoid the elimination and killing of the host immune system and ensure its survival in macrophages for a long time (4, 5). These problems have made *M. tuberculosis* infection a severe health concern in recent years.

The immune system initiates effective defense mechanisms, including cellular and humoral factors, when the host is attacked by pathogens such as bacteria, fungi and viruses (6). While proteins and their immunomodulatory properties have been extensively studied, the roles of noncoding RNAs (ncRNAs) in controlling host defense have not been completely elucidated (especially for *M. tuberculosis* infection).With the development of sequencing technology, a large number of ncRNA species have been discovered (7). NcRNAs are classified into small ncRNAs less than 200 nt in length (including microRNAs (miRNAs), small interfering RNAs (siRNAs), small nuclear RNAs (snRNAs), PIWI-interacting RNAs (piRNAs), long noncoding RNAs (lncRNAs) and circular RNAs (circRNAs) greater than 200 nt in size (7).

In addition, ncRNA plays a role in controlling host gene expression at the transcriptional and posttranscriptional levels. Preliminary studies on the function of ncRNA in infection have focused on pathogen infections such as viruses, parasites and bacteria (8–10). Furthermore, ncRNAs are involved in the host immune response against infection and play important roles in the complex interactions between the host and *M. tuberculosis*. In the past decade, various reports on ncRNA-mediated regulation of *M. tuberculosis* in hosts have been reviewed (11). Here, we focus on the regulation of host ncRNAs involved in host-*M. tuberculosis* interactions.

## Host Cell microRNA Involved in *M. tuberculosis* Infection

### Background of miRNA

As key players in multiple biological processes, microRNAs (miRNAs) play crucial roles in shaping cell differentiation and biological development (12). Dysregulation of miRNA expression will cause diseases, including infection, cancer and immune disorders (9, 13, 14). Navarro and colleagues first found that miRNAs participate in regulating bacterial infection and showed that *Arabidopsis thaliana* recognition of *Pseudomonas syringa* flagellin-derived peptides induces miR-393a transcription and subsequently inhibits the expression of three F-box auxin receptors (15). Taganov et al. showed that miR-146 regulates the immune response by controlling Toll-like receptor 4 (TLR4) and cytokine signaling in monocyte ThP-1 cells induced by lipopolysaccharide (LPS) to protect host cells from excessive inflammation in an NF-κB dependent manner (16). Because each miRNA can regulate hundreds of genes, the dysregulated expression of host miRNAs can affect its vast target gene regulatory network. Subsequent studies established miRNA regulation upon bacterial infection (including *M. tuberculosis*) as a common phenomenon, with implications for multiple host cell functions ranging from autophagy and modulation of immune responses involved in signaling pathways, cell cycle and cell apoptosis. Therefore, a review of the roles of host miRNAs in *M. tuberculosis* infection is particularly important for revealing the pathogenesis of TB and finding anti-tuberculosis drug targets. Next, we summarize host miRNA regulation in the context of *M. tuberculosis* infection.

### The miRNA-Mediated Regulation of Signaling Pathways During *M. tuberculosis* Infection

The miRNAs regulate NF-κB activation induced by TLRs by targeting adaptor proteins in the pathway (17) (**Table 1**). TLR4 is essential for the survival of *M. tuberculosis* infection, while this pathway needs to be controlled to preventing a strong damaging response. Several lines of reports have revealed miRNAs have emerged as important controllers of TLRs signaling (17). For instance, overexpression of miR-708-5p and miR-1178 negatively regulates the level of TLR4, reducing the secretion of proinflammatory factors including interleukin-6 (IL-6), interferon-γ (IFN-γ), interleukin-1β (IL-1β), and tumor necrosis factor-α (TNF-α), thus protecting the host and allowing *M. tuberculosis* contained (18, 19). In RAW264.7 and THP-1 cells infected by *M. tuberculosis*, miR-125a was significantly upregulated in a TLR4 signaling-dependent manner, and then the upregulated miR-125a negatively regulates the NF-κB pathway by directly targeting TRAF6, thereby inhibiting cytokines, attenuating the immune response and promoting *M. tuberculosis* survival in macrophages (20). In infected macrophages, the elimination of *M. tuberculosis* requires a proper immune response; however, an abnormal inflammatory response may lead to the spread of the pathogen (31). MiR-27b is a good example of an ncRNA preventing excessive inflammation and maintaining proinflammatory mediator levels. The TLR2/MyD88/NF-κB pathway triggered by *M. tuberculosis* induces the expression of miR-27b, which inhibits the activity of NF-κB and proinflammatory genes and increases p53 by directly targeting the Bag2 activity of the ROS signaling pathway to positively regulate cell apoptosis (21). *M. tuberculosis* secretes an effector antigen, the early secreted antigenic target 6 (ESAT-6), which downregulates miR-Let-7f in macrophages that targets A20, a feedback inhibitor of the NF-κB pathway. The experiment proved that in A20-deficient macrophages, the production of proinflammatory factors (TNF-α, IL-1β) was increased and *M. tuberculosis* survival was attenuated (22). In infected macrophages, experiments confirmed that Rv2346c, an ESAT-6-like protein, which augmented the phosphorylation of P38, simultaneously upregulates miR-155 and miR-99b, which reduced the production of TNF-α and IL-6 by inhibiting the activation of NF-κB, thereby facilitates intracellular *M. tuberculosis* survival (23).

**Table 1:** MiRNA-mediated regulation of signaling pathways during *M. tuberculosis* infection.

MiRNA	Regulation (Express)		MiRNA-target predictions and validation platform/assay	Predicted targets	Cell types	Outcome	Reference
miR-1178		↑(High)	Bioinformatics analysis and luciferase reporter assay	TLR-4	THP-1 and U937 cells	Inhibit the expression of IFN-γ, IL-6, IL-1β and TNF-α	(18)
miR-708-5p		↑(High)	TargetScan bioinformatics software	TLR-4	THP-1 and U937 cells	Inhibit the expression of IFN-γ, IL-6, IL-1β and TNF-α	(19)
miR-125a		↑(High)	TargetScan bioinformatics software and luciferase reporter assay	TRAF6	RAW264.7 and THP-1 cells	Inhibit the expression of TNF-α, IL-6, IFN-γ and IL-1β	(20)
miR-27b		↑(High)	miRanda、TargetScan、PicTar bioinformatics software and luciferase reporter assay	TLR2	HEK293T and RAW264.7 cells	Inhibit the expression IL-1β,TNF-α,iNOS NF-κB and IL-6	(21)
miR-let-7f		↓(High)	MAGIA bioinformatics software and luciferase reporter assay	A20	RAW264.7,HEK293T,BMDMs, and human MDMs	Reduced production of IL-1β, TNF-α, and NO	(22)
miR-99b		↑(High)	Western blotting 、qRT-PCR analyses and Dual-luciferase reporter assay	P38	U937 and RAW264.7 cells	Promote the expression TNF-α and IL-6	(23)
miR-132		↑(High)	miRWalk bioinformatics software	p300	MDM	Inhibition of IFN-γ signaling cascade	(24)
miR-26a		↑(High)	miRWalk bioinformatics software	p300	MDM	Inhibition of IFN-γ signaling cascade	(24)
miR-20b		↓(High)	Plasmid transfection and luciferase reporter assay	NLRP3	HEK-293T cells	Promote the expression of IL-1β and IL-18, aggravate inflammation	(25)
miR-26a-5p		↓(High)	TargetScan and PicTar bioinformatics software and luciferase reporter assay	CREB-CCEBPβ	RAW264.7 cells	Increased arginase and decreased iNOS activity	(26)
miR-146a		↑(High)	TargetScan bioinformatics software and Plasmid constructs	TRAF6	RAW264.7 cells, Murine BMDMs	Inhibit the expression of iNOS and NO	(27)
miR-21		↑(High)	TargetScan and PicTar bioinformatics software and luciferase reporter assay	IL-12p35	RAW264.7, HEK293T and THP-1 cells	Inhibits IL-12 production and attenuate T cell response	(28)
miR-155		↑(High)	Western blotting analyses and luciferase reporter assay	Bach1, Cox-2 and IL-6	RAW264.7 cells and Murine BMDMs	Promote the activation of Mtb dormancy regulon and attenuate host immune response	(29)
miR-155		↑(High)	cMonkey biclustering algorithm and western blotting analyses	SHIP1	RAW264.7 cells and Murine BMDMs	Promote the activation of Mtb dormancy regulon and attenuate host immune response	(30)

BMDM, bone marrow-derived macrophages; MDM, monocyte derived macrophages; ↑, upregulation; ↓, Downregulation.

**Figure 1:**
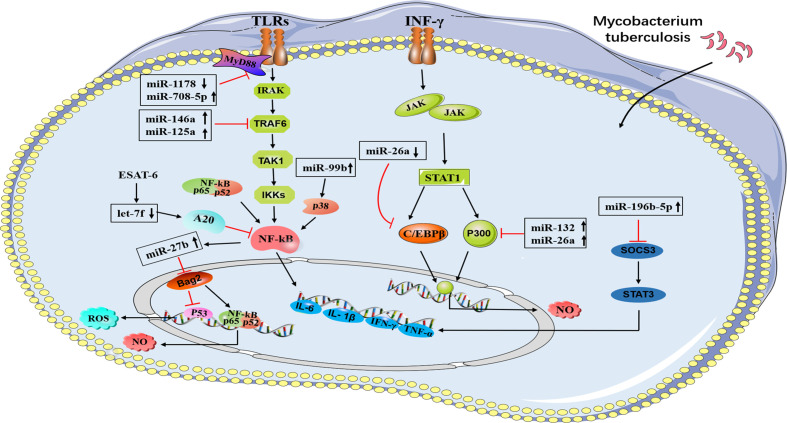
A brief summary of miRNA regulation of signaling pathways during *M. tuberculosis* infection. A schematic diagram represents different miRNAs and their target genes. MiR-708-5p and miR-1178 negatively regulates the level of TLR4 in macrophages. MiR-27b are induced by TLR2/MyD88/NF-κB pathway. MiR-125a and miR146a negatively regulate the NF-κB pathway by directly targeting TRAF6. MiR-132 and miR-26a down-regulate the transcriptional coactivator p300 (a molecule involved in IFN-γ signaling), miR-Let-7f targets A20, a feedback inhibitor of the NF-κB pathway. MiR-99b inhibit the activation of NF-κB, miR-196b-5p activate STAT3 signaling pathway *via* targeting negative regulators SOCS3. 
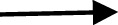
 Direct stimulatory modification; 
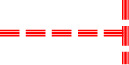
 Direct inhibitory modification.

It is well known that IFN-γ is the predominant activator of macrophage microbicidal activity and IFN-γ-activated macrophages play a key role in fighting intracellular pathogens (24). MiR-132 and miR-26a are upregulated in macrophages upon *M. tuberculosis* infection, which downregulate the transcriptional coactivator p300, a molecule involved in IFN-γ signaling, limiting macrophage response to IFN-γ (24). For efficient clearance of *M. tuberculosis*, macrophages tilt toward M1 polarization, at the same time, the regression of inflammation is related to M2 polarization. MiR-20b is downregulated in *M. tuberculosis*-infected macrophages and directly regulates NLRP3. Further experiments proved that miR-20b induces M1 to M2 macrophage polarization *via* targeting the NLRP3/caspase-1/IL-1β axis (25). In addition, *M. tuberculosis* infection decreases miR-26a-5p, accompanied by upregulation of transcription factor KLF4, and targets CREB-C/EBPβ signaling transduction, which favors M2 macrophage polarization (26). Another study demonstrated that the upregulation of miR-196b-5p could activate the STAT3 signaling pathway *via* targeting negative regulators SOCS3, whereas STAT3 directly affects M2 macrophage polarization, which in turn leads to inhibition of *bacillus Calmette-Guérin* (BCG) uptaken by macrophages due to attenuated proinflammatory responses (32). Furthermore, miR-146a attenuates the activation of NF-κB and mitogen-activated protein kinase signaling pathways during BCG infection, which in turn represses iNOS expression. Moreover, miR-146a modulates the host defense against *M. tuberculosis* infection by repressing NO production by targeting TRAF6 (27) (**Figure 1**).

**Figure 2:**
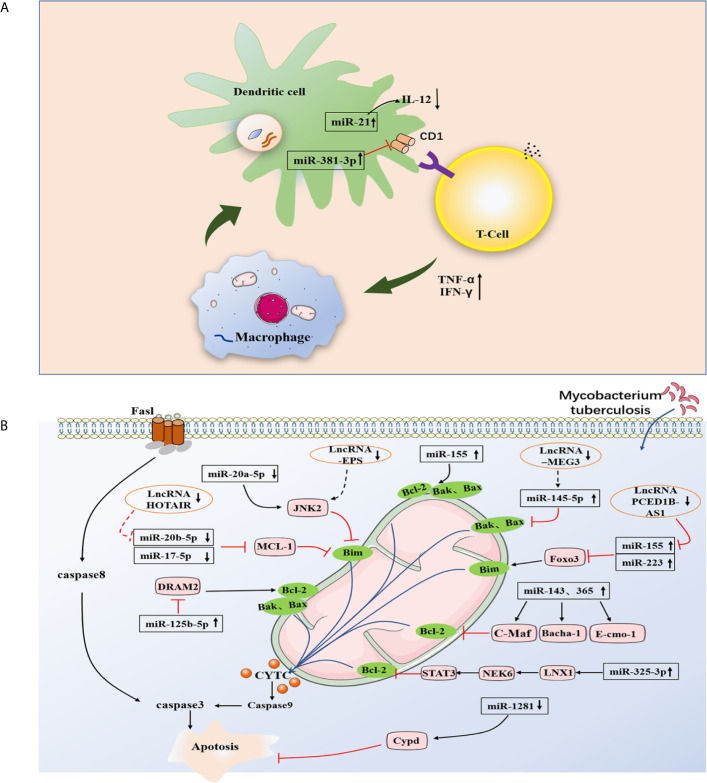
Strategy of non-coding RNA regulating apoptosis pathway in *M. tuberculosis* infected cells. **(A)** MiR-155 and miR223 inhibit apoptosis by targeting FOXO3, miR-1281 inhibited apoptosis by targeting cyclophilin-d, miRNA-143 and miRNA-365 inhibit apoptosis by differentially targeting c-Maf, Bach-1, and Elmo-1. MiR-20a-5p negatively modulating Bim expression in a JNK2-dependent manner. MiR-125b-5p target DRAM2 to promot apoptosis, miR-325-3p targets LNX1 (the E3 ubiquitin ligase of NEK6), leading to abnormal accumulation of NEK6, which in turn activates the STAT3 signaling pathway. At the same time, potential ceRNAs are also flagged here. lincRNA-EPS inhibited apoptosis and enhanced autophagy by activating the JNK/MAPK signaling pathway. PCED1B-AS1 can directly bind to miR-155 to reduce the rate of apoptosis. LncRNA MEG3 can control miR-145-5p expression and regulate macrophage proliferation. The mechanism of action of ceRNA needs to be further studied and verified. **(B)** The apoptotic cells present antigen to DCs to trigger T-cell immunity. MiR-381-3p mediate the reduction of CD1c expression, thereby inhibiting T cell immune responses to *M. tuberculosis.* MiR-21 promotes the apoptosis of DCs by targeting Bcl-2, and inhibits IL-12 production by targeting IL-12p35, weakening the T-cell response to *M. tuberculosis*. 
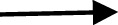
 Direct stimulatory modification; 
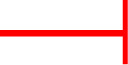
 Direct inhibitory modification; 
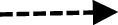
 Tentative stimulatory modification; 
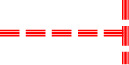
 Tentative inhibitory modification.

The role of miRNA in regulating the immune response of dendritic cells (DCs) against *M. tuberculosis* infection has also been gradually discovered. In DCs with *M. tuberculosis* infection, miR-381-3p is thought to mediate the reduction of CD1c expression, thereby inhibiting T-cell immune responses to *M. tuberculosis* (33). In addition, miR-99b was highly expressed in DCs infected with *M. tuberculosis* strain H37Rv and upregulated the expression of inflammatory factors such as IL-6, IL-12, and IL-1β. More importantly, it regulates the production of TNF-α and TNF-4, thereby activating DCs to clear the phagocytic *M. tuberculosis* (34). After BCG vaccination injection, miR-21 (induced by activated NF-κB) promotes the apoptosis of DCs by targeting Bcl-2 and inhibits IL-12 production by targeting IL-12p35, which weakens the T-cell response to *M. tuberculosis* (28) (**Figure 2A**).

### The miRNA-Mediated Regulation of Apoptosis During *M. tuberculosis* Infection

Evidence have indicated apoptosis of infected macrophages leads to innate control of early bacterial growth in pulmonary *M. tuberculosis* infection (35) (**Figure 2A**). Study also showed apoptotic vesicles from mycobacteria-infected macrophages stimulate CD8 T cells and enhance host control of infection (36).

The involvement of several miRNAs in apoptosis after *M. tuberculosis* infection has also surfaced (**Table 2**). Downregulation of miR-20a-5p was demonstrated to negatively modulate Bim expression in a JNK2-dependent manner and to promote mycobacterial clearance and reduce survival in macrophages, while JNK2 was shown to be a novel direct target of miR-20a-5p (37). In addition, a study found that miR-125b-5p was upregulated in patients with TB and in macrophages infected with *M. tuberculosis* (38). Inhibition of miR-125b-5p can improve the apoptosis rate of macrophages and the expression of apoptosis-related genes Bax and Bim and reduce the secretion of proinflammatory cytokines IL-6 and TNF-α to protect macrophages from injury induced by *M. tuberculosis*. In addition, downregulation of miR-125b-5p also attenuated *M. tuberculosis* infection in human macrophages *in vitro* by targeting DNA damage-regulated autophagy modulator 2 (DRAM2), promoting apoptosis and inhibiting inflammatory response (38). Not surprisingly, *M. tuberculosis* can regulate the host’s miRNAs to inhibit apoptosis, thereby replicating in the cell, conducive to their own survival. Several miRNAs that were differentially expressed were investigated to suppress the apoptosis of *M. tuberculosis*-infected macrophages. In particular, miR-223 in macrophages infected with *M. tuberculosis* inhibits apoptosis by reducing the expression of the transcription factor FOXO3 (39). Similarly, miR-1281 can protect human macrophages from programmed necrosis and apoptosis induced by *M. tuberculosis* by targeting cyclophilin-d (43). Studies have suggested that miRNA-143 and miRNA-365 differentially target c-Maf, Bach-1, and Elmo-1 by promoting the intracellular growth of *M. tuberculosis* in macrophages activated by IL-4/IL-13 (40). The downregulation of miR-20b-5p enhanced *M. tuberculosis* survival in macrophages *via* attenuating the cell apoptosis by Mcl-1 upregulation (44). Recent reports have suggested that miR-325-3p is upregulated after *M. tuberculosis* infection and directly targets LNX1 (the E3 ubiquitin ligase of NEK6), leading to abnormal accumulation of NEK6, which in turn activates the STAT3 signaling pathway, thereby inhibiting apoptosis and promoting the intracellular survival of *M. tuberculosis* (45). Furthermore, miRNAs can also be used in new strategies to regulate host cell phagocytosis; in particular, miR-142-3p, which is induced in *M. tuberculosis*-infected macrophages, can target N-WASP (an actin-binding protein essential for phagocytosis) to regulate the production of phagosomes and reduce the uptake of *M. tuberculosis* (46). Overall, these studies highlight the host cellular miRNA regulation of apoptosis during *M. tuberculosis* infection (**Figure 2B**).

**Table 2:** MiRNA-mediated regulation of apoptosis during *M. tuberculosis* infection.

MiRNA	Regulation (Express)	MiRNA-target predictions and validation platform/assay	Predicted targets	Cell types	Outcome	Reference
miR-27b	↑(High)	miRanda、TargetScan、PicTar bioinformatics software and luciferase reporter assay	Bag2	RAW264.7 and HEK293T cells	Promote the expression of p53 and ROS	(21)
miR-21	↑(High)	TargetScan and PicTar bioinformatics software and luciferase reporter assay	Bcl-2	RAW264.7, HEK293T and THP-1 cells	Promote apoptosis	(28)
miR-20a-5p	↓(High)	RT-PCR analyse and Cells transfection and dual luciferase reporter assay	JNK2	Human macrophages, THP-1 cells and RAW 264.7 cells	Promote Bim expression	(37)
miR-125b-5p	↑(High)	TargetScan bioinformatics software and dual luciferase reporter assay	DRAM2	RAW264.7 and BMDMs	promote apoptosis	(38)
miR-223	↑(High)	Systematic bioinformatics and and Western blot analysis	FOXO3	MDMs and THP-1 cells	Inhibit apoptosis	(39)
miR-143	↑(High)	IRNdb、TargetScan bioinformatics software and luciferase reporter assay	c-Maf, Bach-1 Elmo-1	BMDMs MDMs	Inhibit apoptosis	(40)
miR-365	↑(High)	RNdb、TargetScan bioinformatics software and luciferase reporter assay	c-Maf, Bach-1	BMDMs MDMs	Inhibit apoptosis	(40)
miR-155	↑(High)	Lentivirus-mediated miR-155 sponge and SOCS1 overexpressionl、 Western blotting 、qRT-PCR analyse	SOCS1	RAW264.7 cells	Promote caspase-3 activity	(41)
miR-155	↑(High)	Western blot analysis and luciferase assay	FOXO3	THP-1 cells	Inhibit apoptosis	(42)

BMDM, bone marrow-derived macrophages; MDM, monocyte-derived macrophages; ↑, upregulation; ↓, Downregulation.

### The miRNA-Mediated Regulation of Autophagy During *M. tuberculosis* Infection

**Figure 3:**
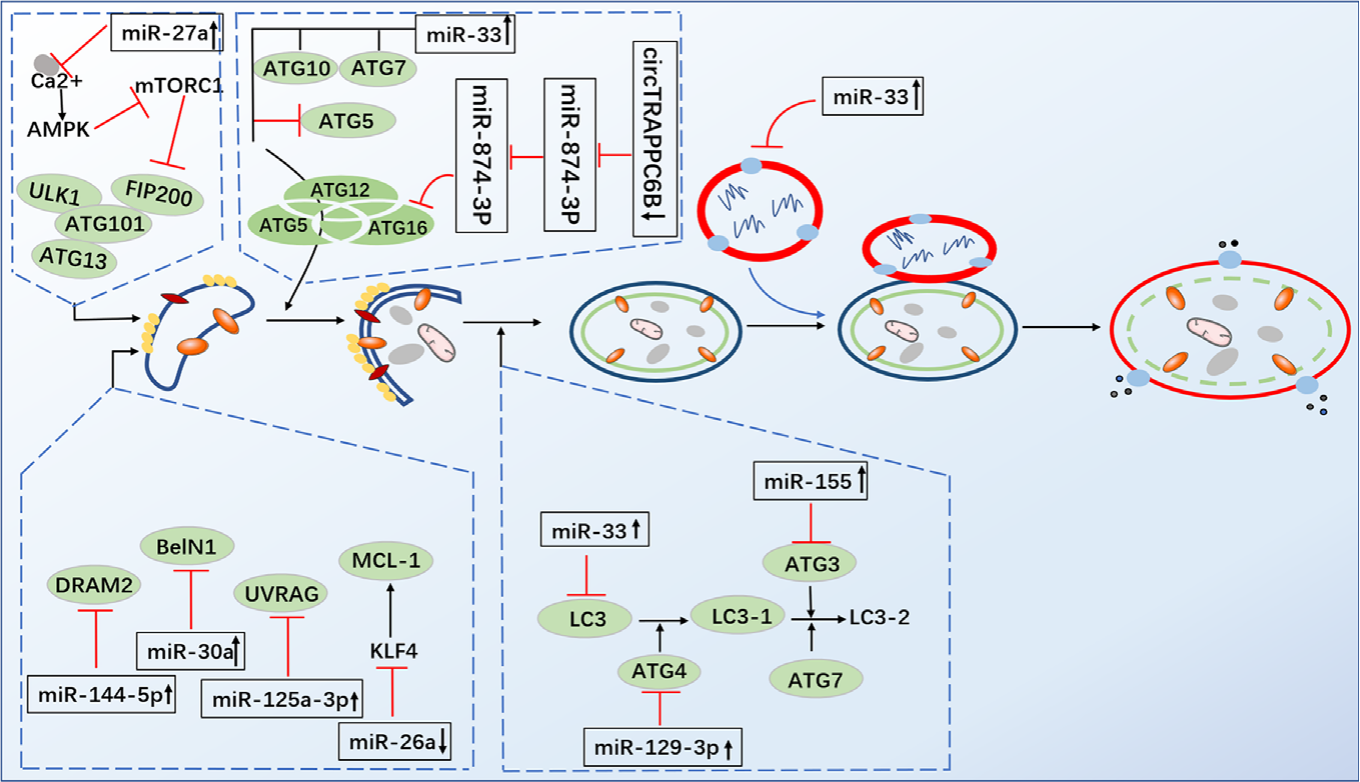
Infection by *M. tuberculosis* leads to alterations of miRNA expression in host cells, which regulate multiple steps of autophagy. MiR-26a facilitates upregulation of the KLF4, that favor the increased expression of Mcl-1 which in turn inhibits autophagosome formation miRNA-17-5p inhibits autophagy by inhibiting Mcl-1 and by binding to Beclin-1 to targe Mcl-1. MiR-33 inhibit autophagic flux by targeting lysosomal pathway transcription factors (FOXO3 and TFEB), activators (AMPK) and multiple effectors (ATG5, ATG12, LC3B and LAMP1). MiR-30a, miR-125a-3p and miR-144-5p respectively targeting Beclin-1, UVRAG and DRAM2. MiR-129-3p inhibit phagosome formation by targeting Atg4b. MiR-27a directly targets the Ca^2+^ transporter Cacna2d3 to inhibit autophagy. CircTRAPPC6B antagonized the ability of miR-874-3p to inhibit ATG16L1 expression, thereby activating and increasing autophagy. 
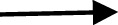
 Direct stimulatory modification; 
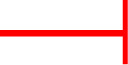
 Direct inhibitory modification; 
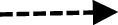
 Tentative stimulatory modification; 
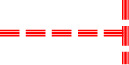
 Tentative inhibitory modification.

Autophagy plays a critical role in maintaining homeostasis within cells. A study in 2004 revealed that autophagy exhibited strong antimicrobial activity against invading pathogens (47). Since the role of autophagy against *M. tuberculosis* was first reported, the literature has further confirmed and enriched the findings (47, 48). One study proved that toxic *M. tuberculosis* in macrophages of infected mice and humans could be effectively eliminated by autophagy (47). The downregulation of miR-26a facilitates upregulation of the KLF4 (transcription factors of macrophage polarization) during *M. tuberculosis* infection, which favors the increased expression of Mcl-1 which in turn inhibits autophagosome formation and consequently lysosomal trafficking of *M. tuberculosis* (26). Moreover, miRNA-17-5p prevents *M. tuberculosis* from being eliminated by regulating autophagy in two ways. A study suggested that reducing miRNA-17-5p inhibits autophagy in *M. tuberculosis*-infected macrophages by inhibiting Mcl-1 and binds to Beclin-1 to target Mcl-1 and STAT3 (transcriptional activator of Mcl-1) (49). Another study showed the increased expression of miR-17-5p inhibits autophagosome formation in BCG-infected cells by inhibiting autophagy activating kinase 1 (ULK1) and the autophagosome-related protein LC3I/II, subsequently reducing the ability of the host cells to kill intracellular BCG (50). Ouimet et al. revealed that miR-33 and miR-33* inhibit autophagic flux by targeting lysosomal pathway transcription factors (FOXO3 and TFEB), activators (AMPK) and multiple effectors (ATG5, ATG12, LC3B and LAMP1) during mycobacterial infection to promote *M. tuberculosis* survival and persistence in cells (51). Upregulation of miR-30a, miR-125a-3p and miR-144-5p levels in *M. tuberculosis*-infected macrophages inhibited autophagy by targeting Beclin-1, UVRAG and DRAM2, respectively, thus suppressing the elimination of intracellular *M. tuberculosis* (52–54). Recent studies have found that miR-889 prevents autophagy by posttranscriptionally inhibiting the expression of TWEAK (an activator of AMP-activated protein kinase AMPK) to maintain mycobacterial survival in granulomas  (55). Furthermore, miR-129-3p expression was triggered by *M. tuberculosis* infection, which was found to be related to autophagy, inhibiting phagosome formation by targeting Atg4b, which was shown to promote *M. tuberculosis* survival in macrophages (56). Additionally, miR-27a inhibited autophagy to promote the intracellular survival of *M. tuberculosis* by directly targeting the Ca^2+^ transporter Cacna2d3 and downregulating ER Ca^2+^ signaling (57). *M. tuberculosis* survival is modulated by miR-23a-5p through the TLR2/MyD88/NF-κB pathway and mediated autophagy by targeting TLR2 during *M. tuberculosis* infection (58) (**Figure 3** and **Table 3**).

**Table 3:** MiRNA-mediated regulation of autophagy during *M. tuberculosis* infection.

MiRNA	Regulation(Express)	MiRNA-target predictions and validation platform/assay	Predicted targets	Cell types	Outcome	Reference
miR-21	↑(High)	TargetScan and PicTar bioinformatics software and luciferase reporter assay	Bcl-2	RAW264.7 cell	Inhibit autophagy	(28)
miRN-17-5p	↓(High)	luciferase assay	Mcl-1	RAW264.7, HEK293 cells and murine BMDMs	Inhibit autophagosome formation	(49)
miR-17–5p	↓(High)	luciferase assay	STAT3	RAW264.7, HEK293 cells and murine BMDMs	Inhibit autophagosome formation	(49)
miR-17–5p	↑(High)	miRanda, PicTar and TargetScan bioinformatics software and luciferase reporter assay	ULK1	RAW264.7 and HEK293T cells	Limitation of phagosomes maturation	(50)
miR-33	↑(High)	PCR and 3′ UTR luciferase reporter assays	FOXO3, TFEB	HEK293 and THP-1 cells	Inhibited autophagic flux	(51)
miR-125a-3p	↑(High)	Targetscan、 miRanda bioinformatics software and luciferase reporter assay	UVRAG	RAW264.7 and J774A.1 cell, BMDMs	Inhibit phagosomal maturation	(52)
miR-144-5p	↑(High)	DIANA-microT、 Targetscan、 miRanda bioinformatics software and luciferase reporter assay	DRAM2	HEK293T and THP-1 cells	Inhibit phagosomal maturation	(54)
miR-889	↑(High)	miRanda bioinformatics software and luciferase reporter assay	TWEAK	THP-1 cells, PBMCs from LTBI patients	Inhibit mycobacterial autophagosome maturation	(55)
miR-129-3p	↑(High)	miRDB、 miRanda、 Targetscan bioinformatics software and luciferase reporter assay	ATG4b	RAW264.7 and HEK-293 T cells	Inhibit autophagic flux	(56)
miR-27a	↑(High)	Base alignment approach and luciferase reporter assay	CACNA2D3	HEK293T cells and Raw264.7 cells, Mouse macrophage	Limit autophagosome formation	(57)
miR-155	↑(High)	TargetScan bioinformatics software and luciferase reporter assay	Rheb	RAW264.7 cells	Promote the maturation of phagosomes	(59)

BMDM, bone marrow-derived macrophages; MDM, monocyte-derived macrophages; PBMC: PBMC, peripheral blood mononuclear cells. ↑, upregulation; ↓, Downregulation.

Each miRNA can regulate hundreds of genes; thus, the same miRNA can perform different functions in host defenses. As an important regulator of adaptive and innate immune responses, miR-155 is encoded by the noncoding gene BIC (42). Under different experimental conditions with different mycobacterial strains, the role of miR-155 in regulating *M. tuberculosis* infection remains controversial. Early research revealed that miR‐155 (the most highly upregulated miRNA in the macrophages of *M. tuberculosis* infection) induction depends on the ESAT-6 and attenuates SH2‐containing inositol 5′‐phosphatase (SHIP1) and the transcriptional repressor BTB and CNC homology 1 (Bach1). Bach1 inhibits the transcription of heme oxygenase-1 (HO-1) (the activator of dormant *M. tuberculosis*), while SHIP1 inhibits serine/threonine kinase Akt activation, which reduces the apoptosis rate of infected macrophages and promotes the survival of *M. tuberculosis* in macrophages (29). Rothchild et al. demonstrated that miR-155 regulates SHIP1-Akt signaling pathways in both macrophages and T cells, which have opposite effects on controlling *M. tuberculosis* infection. In addition, miR-155 maintains the survival and proliferation of bacteria in the early stage of infection in macrophages, whereas miR-155 promotes the long-term maintenance of *M. tuberculosis*-specific T cells capable of secreting the effector cytokines required to control infection in the late stage in T cells (30).

This study revealed a critical and dual role for miR-155 in *M. tuberculosis* infection. Previous studies showed that ESAT-6 stimulation induced miR-155 depending on the activation of TLR2/NF-κB, and miR-155 promoted apoptosis of macrophages by targeting the miR-155/SOCS1 pathway, which was conducive to the clearance of *M. tuberculosis* (29, 41). Ghorpade et al. found that upregulated miR-155 activated caspase-3 and induced the expression of Bid, Bim and Bak1 through TLR2-PI3K-PKC-MAPK signal transduction to induce apoptosis of macrophages after BCG infection (60). Further studies showed that miR-155 in monocytes infected with *M. tuberculosis* inhibited apoptosis by reducing the expression of the transcription factor FOXO3 (42). As a negative regulator of intracellular Rheb expression, miR-155 binds to the Ras homolog (Rheb) in the 3’-untranslated region, which accelerates the process of autophagy and eliminates *M. tuberculosis* in macrophages (59). In DCs, miR-155 induced by *M. tuberculosis* negatively regulates ATG3 and impairs LC3 conversion into its lipidated form (LC3-II), thereby affecting the formation of autophagosomes and inhibiting autophagy to maintain survival of *M. tuberculosis* in cells (61). Compared with that in the lungs of wild-type mice, *M. tuberculosis* infection in miR-155 (-/-) mice was significantly increased, which further demonstrated that miR-155 plays a protective role in the host immune response to *M. tuberculosis* infection (62). Taken together, these studies show that autophagy and *M. tuberculosis* are involved in a compensatory relationship. Understanding how miRNAs regulate autophagy provides a new way to control *M. tuberculosis* infection; that is, a TB immunotherapy method can be based on the optimization of the host cell immune function.

## The Role of circRNAs in Anti-TB Immunity

First identified in RNA viruses by electron microscopy in 1976, circRNAs, were later identified as transcripts in the early 1990s (63). As a special class of endogenous ncRNAs, circRNAs have continued to be reported in viruses, plants, and mammals (63–65). Due to the special mechanism of “back-splicing”, circRNA undergoes a cyclization process, resulting in the lack of a typical terminal structure (5’ cap or 3’ polyadenylation), which makes them resistant to exonucleases (66). On the other hand, circRNAs enriched miRNA-binding sites, therefore serving as miRNA sponges (67). Recent studies have reported circRNAs bind with RNA-related proteins and forming RNA protein complexes that act as RNA-binding protein (RBP) sponges (68), and nuclear localized circRNAs function as potent regulators of transcription at the transcription level (69). Continuous studies have shown that circRNAs play crucial roles in various cellular processes such as proliferation, differentiation, apoptosis, and metastasis (66, 70). Simultaneously, a wide range of circRNAs are highly stable and specific to cells and tissues, and circRNAs are highly expressed in the blood and in bodily fluids secreted by various tissues (such as saliva) (71, 72). Thus, the above studies have demonstrated it is reasonable that circular RNA is implicated with multiple types of diseases (73, 74).

**Figure 4:**
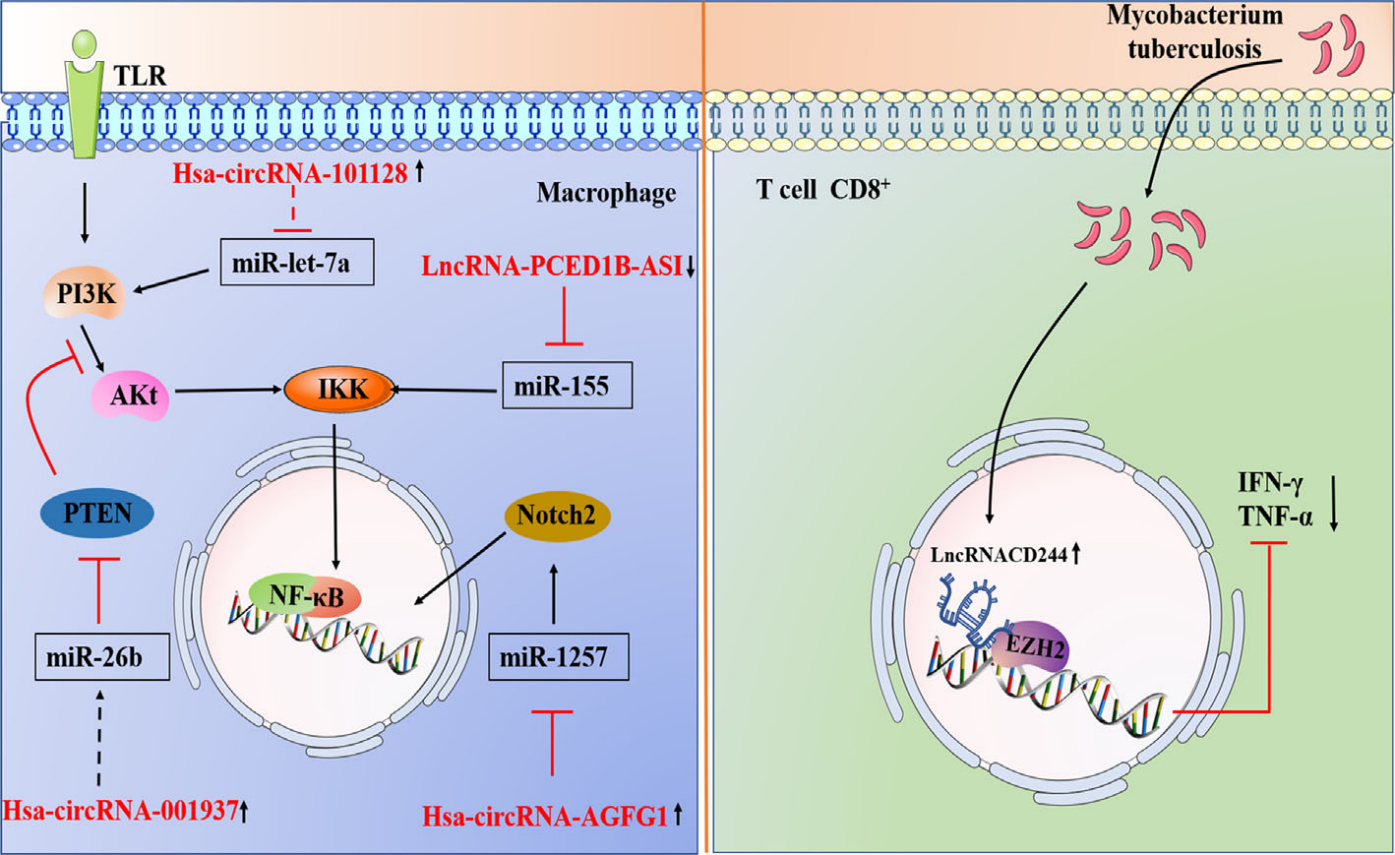
1.A brief summary of circRNA and LncRNA regulation of signaling pathways during *M. tuberculosis* infection. Hsa_circ_101128 may be involved in the pathogenesis of active TB by negatively regulating let-7a and may be involved in the MAPK and PI3K-AKT pathways. Hsa_circ_001937 potential miRNA target is miR-26b, which participate in the inflammatory response by modulating the NF-κB pathway by targeting PTEN. CircAGFG1 can enhance autophagy is achieved by targeting miRNA1257 to regulate Notch signal in the macrophages infected by *M. tuberculosis*. 2.Models involving lncRNAs that target the host immune system of *M. tuberculosis*. *M. tuberculosis* infection leads to upregulation of lncRNA-CD244 in CD8^+^ T cells, which interacts with chromatin modifying enzyme EZH2, and lncRNA-CD244 acts as an epigenetic regulator of IFN-γ and TNF-α in CD8^+^ T cells and suppresses their expression to modulate the TB immune response of CD8^+^ T cells. 
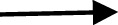
 Direct stimulatory modification; 
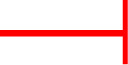
 Direct inhibitory modification; 
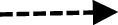
 Tentative stimulatory modification; 
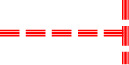
 Tentative inhibitory modification.

A link of circRNAs to infectious disease has also been established. For instance, Wang et al. confirmed that circ-chr19 enhances the expression of CLDN18 (which affects cell permeability) by targeting miR-30b-3p in Ebola virus infection (75). During the host response against viral infection, the immune factor NF90/NF110 exits the nucleus and reduces the expression of circRNA; at the same time, more NF90/NF110 is released through circRNPs and binds to viral mRNAs, exerting an antiviral effect (76). Liu et al. found that circRNA_051239 was significantly upregulated in drug-resistant TB patients, and circRNA_051239 may act as sponges of miR-320a and play a crucial role in the development of TB drug resistance (77, 78). Moreover, circAGFG1 can enhance autophagy and reduce the rate of apoptosis, which is achieved by targeting miRNA1257 to regulate Notch signaling in the macrophages infected by *M. tuberculosis* (79). Another study indicated that in the macrophages infected by *M. tuberculosis*, the overexpression circRNA-0003528 upregulates CTLA4 by downregulating miR-224-5p, miR-324-5p, and miR-488-5p to promote macrophage polarization (80). Recent studies have demonstrated that circTRAPPC6B, as a novel ceRNA, antagonized the ability of miR-874-3p to inhibit ATG16L1 expression, thereby activating and increasing autophagy to limit *M. tuberculosis* growth in macrophages (**Figure 3**) (81). Numerous studies have identified circRNAs that are differentially expressed in *M. tuberculosis* infection and predicted their target miRNAs (**Figure 4** and **Table 4**). Due to the biological characteristics of circRNAs, they have become potential biomarkers of *M. tuberculosis* infection stages (89). Zhuang et al. found that hsa_circ_0005836 and hsa_circ_0009128 in PBMCs of patients with active TB were significantly downregulated and indicated hsa_circ_0005836 might serve as a novel potential diagnostic biomarker for *M. tuberculosis* infection (82). Gene Ontology and KEGG enrichment analyses showed that differential expression of circular RNA was related to immune system activation, which indicated that there is a correlation between *M. tuberculosis* infection and immune system activity (82). Huang et al. discovered that the expression of hsa_circ_0043497 and hsa_circ_0001204 in monocyte-derived macrophages (MDMs) was significantly increased, and they found that the potential target miRNAs may be miR-377-3p and miR-186-5p (83). Subsequently, the team found that hsa_circ_001937 was significantly increased in the PBMCs of TB patients and was associated with TB severity, suggesting that its levels may be related to the clinical grade and stage of TB, and its potential miRNA target is miR-26b (84). Furthermore, miR-26b has been shown to participate in the inflammatory response by modulating the NF-κB pathway by targeting PTEN (90). Fu et al. found that circRNA_103017, circRNA_101128 and circRNA_059914 were increased in TB patients. Bioinformatics analysis indicated that hsa_circ_101128 may be involved in the pathogenesis of active TB by negatively regulating let-7a and may be involved in the MAPK and PI3K-AKT pathways, which are thought to be associated with active TB (85). Zhang et al. found that STAT1 and its related molecules, including hsa-miR-223-3p, hsa-miR-448, SAMD8_hsa_circWF1_hsa_circRNA9897, are potential biomolecules for the host’s defense response to *M. tuberculosis* infection (86). Another study showed that hsa_circ_103571 was significantly reduced in active TB patients and showed potential interactions with TB-associated miRNAs such as miR-29a and miR-16 (87, 88).

**Table 4:** The regulatory role of CircRNAs in anti-TB immunity.

Circular RNA	Regulation(Express)	Samples	Technology of CircRNAs expression	Predicted miRNAs targets	Potential value	Reference
circRNA_051239	↑(High)	Serum	Microarray analysis、RT-qPCR	miR-320a	Be related to drug resistance	(77, 78)
circAGFG1	↑(High)	Bronchoalveolar lavage	Western blotting, Cell transfection, CCK-8 assay, flow cytometry, RT-qPCR, luciferase reporter assay	miRNA-1257	Decreased monocyte apoptosis and enhanced autophagy	(79)
circRNA-0003528	↑(High)	Plasma	RT-qPCR, luciferase reporter assay	miR-224-5p, miR-324-5p, miR-488-5p	Promote macrophage polarization	(80)
circTRAPPC6B	↓(High)	PBMCs	Plasmid transfection, RT-qPCR, Western blot, Bioinformatics prediction, luciferase assay, FISH	miR-874-3p	Enhanced autophagy	(81)
hsa_circ_0005836	↓(High)	PBMCs	High-throughput sequencing, RT-qPCR	hsa-miR-93-3p, hsa-miR-367-5p, hsa-miR-629-3p	Potential biomarker for TB	(82)
has_circ_0009128	↓(High)	PBMCs	High-throughput sequencing, RT-qPCR	hsa-miR-93-3p, hsa-miR-367-5p, hsa-miR-629-3p	Belated to immune system activation	(82)
hsa_circ_0043497	↑(High)	PBMCs	Microarray analysis、RT-qPCR	miR-335-3p,miR-186-5p,miR-380-5p,miR-296-3p,miR-522-3p	Potential biomarker for TB	(83)
hsa_circ_0001204	↓(High)	Plasma specimens	Microarray analysis、RT-qPCR	miR-612,miR-657,miR-362-3p,miR-377-3p,miR-136-5p	Potential biomarker for TB	(83)
hsa_circ_001937	↑(High)	PBMCs	Microarray analysis、RT-qPCR	miR-22-5p, miR-26b-3p, miR-10b-3p	Effective diagnostic biomarkers for TB	(84)
hsa_circ_101128	↑(High)	PBMCs	Microarray analysis、RT-qPCR	let-7a	Be related to autophagy	(85)
SAMD8_hsa_circRNA994	NA	whole blood	GEO database, GSEA,RT-qPCR	hsa-miR-223–3p	Be related to autophagy	(86)
TWF1_hsa_circRNA9897	NA	whole blood	GEO database, GSEA,RT-qPCR	hsa-miR-448	Be related to autophagy	(86)
has_circ_103571	↑(High)	Plasma specimens	Microarray analysis、RT-qPCR	miR-29a,miR-16	Be related to autophagy	(87, 88)

PBMC, peripheral blood mononuclear cells. ↑, upregulation; ↓, Downregulation; NA, not available.

All the above studies have elucidated the presence of differentially expressed circRNAs in *M. tuberculosis*-infected host cells. However, it remains unknown how these circRNAs are involved in the regulation of miRNAs in *M. tuberculosis* infection. To investigate this question, different research groups focused on downstream signaling pathways to analyze differentially expressed circRNAs to identify potential miRNAs with potential binding sites, and further identify potential target genes for these miRNAs. This approach has been used in an attempt to identify candidate circRNAs as novel diagnostic markers, which could provide reliable targets for the treatment of TB. However, more research is still needed to elucidate the biological role of circRNAs in *M. tuberculosis*-host interactions and their true potential as clinical indicators.

## The Role of lncRNAs in Anti-TB Immunity

Over the last decade, tens of thousands of lncRNAs have been discovered in mammalian genomes (91). While lncRNAs constitute a class of RNAs that are longer than 200 nucleotides, they are not translated into a protein product and instead function as an RNA molecule (92). Similar to most mRNAs, lncRNAs have a special cap structure at 5’ and a polyadenylate at 3’, and it was previously thought that there was no open reading frame in their sequence (91, 93). Moreover, lncRNAs can localize to target sites within the nucleus or cytoplasm of the cell and are widely expressed in eukaryotes (94, 95). They vary among different species but have high cell-type specificity (91). However, a few recent studies have confirmed that some lncRNAs show small open reading frames that encode short peptides with key biological functions. The existence of small functional peptides encoded by these lncRNAs indicates that these lncRNAs can play dual roles in RNA and peptides at the same time (96). In addition, lncRNAs are classified according to the relative position of the protein-coding gene, namely, forward lncRNAs, reverse lncRNAs, bidirectional lncRNAs, intragenic lncRNAs, and intergenic lncRNAs (97).

Emerging as an important regulator in many aspects of biology, lncRNAs have been proven to play an important role in various biological processes from development to immune response (98). For example, lncRNA-EPS, an inhibitor of the inflammatory response, is precisely regulated in macrophages to control the expression of immune response genes (IRGs) (99), and lncRNA-cox2 regulates activation and represses immune response genes induced by TLRs (100).

Fortunately, lncRNAs offer a new direction in exploring human host immunity to *M. tuberculosis* infection. A larger number of studies have shown abnormally expressed lncRNAs in macrophages of TB patients by different experimental methods (101–104) (**Figure 2B** and **Table 5**). For instance, the lncRNA HOTAIR facilitates the survival of virulent *M. tuberculosis* in SATB1- and DUSP4-dependent manners (101). Kamlesh Pawar et al. reported that IFN-γ-mediated autophagy in infected macrophages leads to the downregulation of lncRNA MEG3 expression, which contributes to the elimination of intracellular mycobacterium in BCG infection (103). In addition, lncRNAs play a role in transcriptional and posttranscriptional gene regulation (110). LncRNAs enriched miRNA-binding sites, therefore serving as miRNA sponges and competing with the target mRNAs for binding miRNA in the cytoplasm. This indicates that lncRNA regulate gene expression at the posttranscriptional level and participate in RNA networks, acting as competing endogenous RNAs (ceRNAs) (111). The ceRNA regulation hypothesis was proposed by Salmena et al. (112). Recent studies have shown that lncRNA MEG3 can also control miR-145-5p expression and regulate macrophage proliferation to control *M. tuberculosis* infection (105). Ke et al. found that lncRNA-EPS expression was downregulated in monocytes from patients with active pulmonary tuberculosis (PTB) compared with those in healthy individuals. Further research shows that knocking down lncRNA-EPS inhibited apoptosis and enhanced autophagy by activating the JNK/MAPK signaling pathway in BCG-infected RAW264.7 macrophages, allowing *M. tuberculosis* to survive in macrophages (104). Li et al. found that the expression of PCED1B-AS1 was downregulated in patients with active TB, and PCED1B-AS1 acted as an endogenous sponge to block the expression of miR-155 in macrophages by directly binding to miR-155, thereby reducing the apoptosis rate and promoting autophagy (**Figures 2B** and **4**) (106). Bai et al. found that the expression levels of lnc-AC145676.2.1-6 and lnc-TGS1-1 were significantly downregulated in PTB (107). The previous studies and bioinformatics predictions suggest that lnc-TGS1-1and lnc-AC145676.2.1-6 may be able to act as miRNA sponges to interact with miR-143 and miR-29a to participate in the occurrence and development of TB (107). One study investigated the effect of lncRNA NEAT1 (nuclear-rich transcript1) on *M. tuberculosis* infection. Moreover, NEAT1 promoted the increase of inflammatory factors in *M. tuberculosis*-infected macrophages, reduced the phagocytosis of macrophages and inhibited cell apoptosis through regulating miR-377-3p, leading to the occurrence of TB (108). Moreover, ceRNA analysis of ENST00000570366, NR_003142, NR_038221, and ENST00000422183 predicted the potential relationships with ncRNAs. The results indicated that NR_038221 was the most considerably associated with TB (113). A previous study verified hsa-miR-378a-3p as a potential biomarker for pulmonary TB, and hsa-miR-378a-3p was associated with NR_038221, which indicated NR_038221 and hsa-miR-378a-3p might play a similar function during the pathological process of pulmonary TB (113, 114).

**Table 5:** The regulatory role of lncRNAs in anti-TB immunity.

LncRNAs	Regulation(Express)	Technology of LncRNAs expression	Predicted targets	Validated targets	Samples	Function	Reference
LncRNA HOTAIR	↓(High)	Chromatin Immunoprecipitation, western blot, RT-qPCR, gene silencing by siRNA		EZH2	THP-1 cells	Favors the transcription of SATB1 and DUSP4 and inhibit the production of ROS	(101)
LincRNA-EPS	↓(High)	Flow cytometry, RT-qPCR, Immunofluorescence, western blot		JNK/MAPK	RAW264.7 cells	Attenuate apoptosis and enhance autophagy	(104)
LncRNA MEG3	↓(High)	Dual-luciferae reporter assay,Flow cytometry, RT-qPCR	miR-145-5p		THP-1, U937, HeLa, HT-29 cells	Attenuate the ability of inhibiting autophagy	(105)
LncRNA PCED1B-AS1	↓(High)	Microarray analysis, western blot, CCK-8 assay, immunofluorescence and TEM, flow cytometry	miR-155		PBMCs, THP-1 cells	Attenuate in monocyte apoptosis and enhance in autophagy	(106)
Lnc-AC145676.2.1-6	↓(High)	RT-qPCR	miR-29a		Whole blood	Interference with the toll-like receptor signaling pathway and other immune-response interactions	(107)
Lnc-TGS1-1	↓(High)	RT-qPCR	miR-143		Whole blood	Leads to presence of thrombocytopenia during anti-TB treatment/interference with the toll-like receptor signaling pathway and other immune-response interactions	(107)
LncRNA NEAT1	↑(High)	RT-qPCR, gene silencing by siRNA	miR-377-3p		PBMCs	Decrease inIL-6/enhances in duration of infection/related with outcome of TB	(108)
LncRNA-CD244	↑(High)	Flow cytometry, intracellular cytokine staining (ICS), immune analyses of MTB-infected mice		EZH2	CD8^+^ T cells	Inhibit the expression of IFN-γ and TNF-α	(109)

↑, upregulation; ↓, Downregulation.

In the immune response to *M. tuberculosis* infection, CD4^+^ T cell immunity is dominant. CD4^+^ T cells can produce cytokines, which in turn activate macrophages to inhibit the growth of intracellular *M. tuberculosis* (115). Although the role of CD8^+^T cells in the immune response to *M. tuberculosis* infection is controversial, recent studies have confirmed that CD8^+^T cells, similar to CD4^+^T cells, produce the critical functions of IL-2, IFN-γ and TNF, thereby providing a protective immune response after infection with *M. tuberculosis* (115, 116). Zeng’s research group found that lncRNA-CD244 acts as an epigenetic regulator of IFN-γ and TNF-а in CD8^+^ T cells and inhibits their expression to regulate the TB immune response of CD8^+^ T cells (109). Fu et al. found that lncRNA was differentially expressed in CD8^+^ T cells, and heme oxygenase 1 (HMOX1) was downregulated in CD8^+^T cells of PTB patients, while its related lincRNA (XLOC-014219) was upregulated, suggesting that lncRNA may be related to the dysfunction of CD8^+^ T cells and may participate in the pathophysiological process of active PTB (117); in the same year, they also found that SOCS3 (a key negative regulator of the response to *M. tuberculosis* infection) and its adjacent lncRNA (XLOC-012582) were highly expressed in *M. tuberculosis-*infected B cells (118).

In recent years, with the development of modern biotechnology such as gene chips, a larger number of lncRNAs have been continuously discovered, and the functions of lncRNAs and their regulatory mechanisms upon *M. tuberculosis* infection have been studied from many perspectives, particularly their differential expression in host cells. Thus, lncRNAs will help us understand the pathogenesis of TB and provide new clues for the prevention and treatment of TB.

## Conclusions and Perspectives

Overall, the advancement of RNA-sequencing technology has contributed to the discovery of host ncRNAs. Host ncRNA is now considered to be the main participant in the infection process of *M. tuberculosis.* Many studies have revealed differentially expressed ncRNAs in TB patients and healthy individuals, but whether differentially expressed ncRNAs can be used as ideal biomarkers for the diagnosis of TB or as targets for the treatment of TB remains to be determined, and many questions still need to be answered, such as are there sex and race differences for the ncRNAs that are differentially expressed? In addition, understanding whether ncRNA regulates *M. tuberculosis* infection as a common phenomenon or whether specific phenomena exist under certain conditions remains unclear. Additionally, most of the research about ncRNA mainly focuses on miRNA. More research focused on circRNA and lncRNA studies regarding *M. tuberculosis* infection would be of benefit in attracting people’s attention to these areas. Despite the problems and questions, ncRNAs are highly promising as biomarkers of TB. Due to the complexity of the ncRNAs themselves, there are many host ncRNAs that have not been discovered, and their functions and regulatory networks are thus unknowable. In addition, the lack of accurate databases for the host ncRNAs that are being discovered is restricting research to the analysis of differences in gene expression. The corresponding gene regulatory function, the identification of downstream targets and the potential mechanisms involved in regulation still need to be further studied.

In this article, we summarized the role and mechanism of dysregulated expression of ncRNA in regulating host immune response in *M. tuberculosis* infection. Understanding this could promote the development of therapeutic strategies against *M. tuberculosis* infection that are used as therapeutic targets, that is, by reducing or increasing the expression of key ncRNAs and then inhibiting or activating the genes influenced by these ncRNAs. In fact, this ncRNA-based therapeutic approach is currently under development.

Finally, these data will not only provide basic knowledge about the function of ncRNAs in host-*M. tuberculosis* interactions but will also be critical for the development of new anti-TB diagnostic and therapeutic approaches.

## Author Contributions

LW drafted the manuscript. QJ drew the figures. HZ and KL contributed equally to plot the table. BZ and QB revised the review. All authors contributed to the article and approved the submitted version.

## Funding

This study was supported by grants from the National Natural Science Foundation of China (No. 81702439, 81802446), Tai Shan Young Scholar Foundation of Shandong Province (No. tsqn201909192), Shandong Provincial Natural Science Foundation (No. ZR2019BH050, ZR2020YQ59), PhD Research Foundation of the Affiliated Hospital of Jining Medical University (No. 2018-BS-001, 2018-BS-013), the Project of Medicine Health and Technology Development Plan of Shandong Province (NO. 202003031182, 202003031183), Jining Medical University Teacher Research Support Fund (NO. JYFC2018FKJ035), and the Miaopu Research of the Affiliated Hospital of Jining Medical University (No. MP-ZD-2020-005).

## Conflict of Interest

The authors declare that the research was conducted in the absence of any commercial or financial relationships that could be construed as a potential conflict of interest.

## References

[1] World Health Organization. Global Tuberculosis Report. Geneva, Switzerland: World Health Organization (2019).

[2] Wright A Zignol M Van Deun A Falzon D Gerdes SR Feldman K. Epidemiology of Antituberculosis Drug Resistance 2002-07: An Updated Analysis of the Global Project on Anti-Tuberculosis Drug Resistance Surveillance. Lancet (2009) 373(9678):1861–73. 10.1016/s0140-6736(09)60331-7 19375159

[3] MacDonald EM Izzo AA. Tuberculosis Vaccine Development — Its History and Future Directions. In: Tuberculosis - Expanding Knowledge, IntechOpen (2015) 10.5772/59658

[4] Stanley SA Cox JS. Host-Pathogen Interactions During Mycobacterium Tuberculosis Infections. Curr Top Microbiol Immunol (2013) 374:211–41. 10.1007/82_2013_332 23881288

[5] Guirado E Schlesinger LS Kaplan GJ. Macrophages in Tuberculosis: Friend or Foe. Semin Immunopathol (2013) 35(5):563–83. 10.1007/s00281-013-0388-2 PMC3763202 23864058

[6] Eulalio A Schulte L Vogel J. The Mammalian microRNA Response to Bacterial Infections. RNA Biol (2012) 9(6):742–50. 10.4161/rna.20018 22664920

[7] Mcfadden EJ Hargrove AE. Biochemical Methods to Investigate lncRNA and the Influence of LncRNA:Protein Complexes on Chromatin. Biochemistry (2015) 55(11):1615–30. 10.1021/acs.biochem.5b01141 PMC5010801 26859437

[8] Bruscella P Bottini S Baudesson C Pawlotsky JM Feray C Trabucchi M. Viruses and MiRNAs: More Friends Than Foes. Front Microbiol (2017) 08:824. 10.3389/fmicb.2017.00824 PMC5430039 28555130

[9] Maudet C Mano M Eulalio A. MicroRNAs in the Interaction Between Host and Bacterial Pathogens. FEBS Lett (2014) 588(22):4140–7. 10.1016/j.febslet.2014.08.002 25128459

[10] Zheng Y Cai X Bradley JE. MicroRNAs in Parasites and Parasite Infection. RNA Biol (2013) 10(3):371–9. 10.4161/rna.23716 PMC3672280 23392243

[11] Arnvig K Young D. Non-Coding RNA and its Potential Role in Mycobacterium Tuberculosis Pathogenesis. RNA Biol (2012) 9(4):427–36. 10.4161/rna.20105 PMC3384566 22546938

[12] Shenoy A Blelloch RH. Regulation of microRNA Function in Somatic Stem Cell Proliferation and Differentiation. Nat Rev Mol Cell Biol (2014) 15(9):565–76. 10.1038/nrm3854 PMC4377327 25118717

[13] Lee YS Dutta A. MicroRNAs in Cancer. Annu Rev Pathol (2009) 4:199–227. 10.1146/annurev.pathol.4.110807.092222 18817506 PMC2769253

[14] Sonkoly E Pivarcsi A. MicroRNAs in Inflammation. Int Rev Immunol (2009) 28(6):535–61. 10.3109/08830180903208303 19954362

[15] Lionel N. A Plant miRNA Contributes to Antibacterial Resistance by Repressing Auxin Signaling. J Sci (New York NY) (2006) 5772(312):436–9. 10.1126/science.1126088 16627744

[16] Taganov KD Boldin MP Chang KJ Baltimore D. NF-kappaB-dependent Induction of microRNA miR-146, an Inhibitor Targeted to Signaling Proteins of Innate Immune Responses. Proc Natl Acad Sci U S A (2006) 103(33):12481–6. 10.1073/pnas.0605298103 PMC1567904 16885212

[17] O’Neill LA Sheedy FJ McCoy CE. MicroRNAs: The Fine-Tuners of Toll-like Receptor Signalling. Nat Rev Immunol (2011) 11(3):163–75. 10.1038/nri2957 21331081

[18] Shi G Mao G Xie K Wu D Wang W. MiR-1178 Regulates Mycobacterial Survival and Inflammatory Responses in Mycobacterium Tuberculosis-Infected Macrophages Partly Via TLR4. J Cell Biochem (2018) 119(9):7449–57. 10.1002/jcb.27054 29781535

[19] Li WT Zhang Q. MicroRNA-708-5p Regulates Mycobacterial Vitality and the Secretion of Inflammatory Factors in Mycobacterium Tuberculosis-Infected Macrophages by Targeting TLR4. Eur Rev Med Pharmacol Sci (2019) 23(18):8028–38. 10.26355/eurrev_201909_19019 31599428

[20] Niu W Sun B Li M Cui J Huang J Zhang L. TLR-4/MicroRNA-125a/NF-κB Signaling Modulates the Immune Response to Mycobacterium Tuberculosis Infection. Cell Cycle (2018) 17(15):1931–45. 10.1080/15384101.2018.1509636 PMC6152532 30153074

[21] Liang S Song Z Wu Y Gao Y Gao M Liu F. MicroRNA-27b Modulates Inflammatory Response and Apoptosis During Mycobacterium Tuberculosis Infection. J Immunol (2018) 200(10):3506–18. 10.4049/jimmunol.1701448 29661829

[22] Kumar M Sahu SK Kumar R Subuddhi A Maji RK Jana K. MicroRNA Let-7 Modulates the Immune Response to Mycobacterium Tuberculosis Infection Via Control of A20, an Inhibitor of the NF-κB Pathway. Cell Host Microbe (2015) 17(3):345–56. 10.1016/j.chom.2015.01.007 25683052

[23] Yao J Du X Chen S Shao Y Deng K Jiang M. Rv2346c Enhances Mycobacterial Survival Within Macrophages by Inhibiting TNF-α and IL-6 Production Via the P38/MiRNA/NF-κB Pathway. Emerg Microbes Infect (2018) 7(1):158. 10.1038/s41426-018-0162-6 30232332 PMC6145905

[24] Ni B Rajaram MV Lafuse WP Landes MB Schlesinger LS. Mycobacterium Tuberculosis Decreases Human Macrophage IFN-γ Responsiveness Through miR-132 and miR-26a. J Immunol (2014) 193(9):4537–47. 10.4049/jimmunol.1400124 25252958

[25] Lou J Wang Y Zhang Z Qiu W. MiR-20b Inhibits Mycobacterium Tuberculosis Induced Inflammation in the Lung of Mice Through Targeting NLRP3. Exp Cell Res (2017) 358(2):120–8. 10.1016/j.yexcr.2017.06.007 28606793

[26] Sahu SK Kumar M Chakraborty S Banerjee SK Kumar R Gupta P. MicroRNA 26a (miR-26a)/KLF4 and CREB-C/EBPβ Regulate Innate Immune Signaling, the Polarization of Macrophages and the Trafficking of Mycobacterium Tuberculosis to Lysosomes During Infection. PloS Pathog (2017) 13(5):e1006410. 10.1371/journal.ppat.1006410 28558034 PMC5466338

[27] Li M Wang J Fang Y Gong S Li M Wu M. MicroRNA-146a Promotes Mycobacterial Survival in Macrophages Through Suppressing Nitric Oxide Production. Sci Rep (2016) 6:23351. 10.1038/srep23351 27025258 PMC4812255

[28] Wu Z Lu H Sheng J Li L. Inductive microRNA-21 Impairs Anti-Mycobacterial Responses by Targeting IL-12 and Bcl-2. FEBS Lett (2012) 586(16):2459–67. 10.1016/j.febslet.2012.06.004 22710123

[29] Kumar R Halder P Sahu SK Kumar M Kumari M Jana K. Identification of a Novel Role of ESAT-6-dependent miR-155 Induction During Infection of Macrophages With Mycobacterium Tuberculosis. Cell Microbiol (2012) 14(10):1620–31. 10.1111/j.1462-5822.2012.01827.x 22712528

[30] Rothchild AC Sissons JR Shafiani S Plaisier C Min D Mai D. MiR-155-regulated Molecular Network Orchestrates Cell Fate in the Innate and Adaptive Immune Response to Mycobacterium Tuberculosis. Proc Natl Acad Sci U S A (2016) 113(41):E6172–e6181. 10.1073/pnas.1608255113 27681624 PMC5068277

[31] ScribaTJCoussensAKFletcherHA. Human Immunology of Tuberculosis. Microbiol Spectr (2017) 5(1):213–37. 10.1128/microbiolspec.TBTB2-0016-2016 28155806

[32] Yuan Y Lin D Feng L Huang M Yan H Li Y. Upregulation of miR-196b-5p Attenuates BCG Uptake Via Targeting SOCS3 and Activating STAT3 in Macrophages From Patients With Long-Term Cigarette Smoking-Related Active Pulmonary Tuberculosis. J Transl Med (2018) 16(1):284. 10.1186/s12967-018-1654-9 30326918 PMC6192289

[33] Wen Q Zhou C Xiong W Su J He J Zhang S. MiR-381-3p Regulates the Antigen-Presenting Capability of Dendritic Cells and Represses Antituberculosis Cellular Immune Responses by Targeting CD1c. J Immunol (2016) 197(2):580–9. 10.4049/jimmunol.1500481 27296666

[34] Singh Y Kaul V Mehra A Chatterjee S Tousif S Dwivedi VP. Mycobacterium Tuberculosis Controls microRNA-99b (miR-99b) Expression in Infected Murine Dendritic Cells to Modulate Host Immunity. J Biol Chem (2013) 288(7):5056–61. 10.1074/jbc.C112.439778 PMC3576108 23233675

[35] Behar SM Martin CJ Booty MG Nishimura T Zhao X Gan HX. Apoptosis is an Innate Defense Function of Macrophages Against Mycobacterium Tuberculosis. Mucosal Immunol (2011) 4(3):279–87. 10.1038/mi.2011.3 PMC3155700 21307848

[36] Winau F Weber S Sad S de Diego J Hoops SL Breiden B. Apoptotic Vesicles Crossprime CD8 T Cells and Protect Against Tuberculosis. Immunity (2006) 24(1):105–17. 10.1016/j.immuni.2005.12.001 16413927

[37] Zhang G Liu X Wang W Cai Y Li S Chen Q. Down-Regulation of miR-20a-5p Triggers Cell Apoptosis to Facilitate Mycobacterial Clearance Through Targeting JNK2 in Human Macrophages. Cell Cycle (2016) 15(18):2527–38. 10.1080/15384101.2016.1215386 PMC5026815 27494776

[38] Liu G Wan Q Li J Hu X Gu X Xu S. Silencing miR-125b-5p Attenuates Inflammatory Response and Apoptosis Inhibition in Mycobacterium Tuberculosis-Infected Human Macrophages by Targeting DNA Damage-Regulated Autophagy Modulator 2 (DRAM2). Cell Cycle (2020) 19(22):3182–94. 10.1080/15384101.2020.1838792 PMC7714508 33121314

[39] Xi X Zhang C Han W Zhao H Zhang H Jiao J. MicroRNA-223 Is Upregulated in Active Tuberculosis Patients and Inhibits Apoptosis of Macrophages by Targeting Foxo3. Genet Test Mol Biomarkers (2015) 19(12):650–6. 10.1089/gtmb.2015.0090 26505221

[40] Tamgue O Gcanga L Ozturk M Whitehead L Pillay S Jacobs R. Differential Targeting of C-Maf, Bach-1, and Elmo-1 by microRNA-143 and MicroRNA-365 Promotes the Intracellular Growth of Mycobacterium Tuberculosis in Alternatively Il-4/Il-13 Activated Macrophages. Front Immunol (2019) 10:421. 10.3389/fimmu.2019.00421 30941122 PMC6433885

[41] Yang S Li F Jia S Zhang K Jiang W Shang Y. Early Secreted Antigen ESAT-6 of Mycobacterium Tuberculosis Promotes Apoptosis of Macrophages Via Targeting the microRNA155-SOCS1 Interaction. Cell Physiol Biochem (2015) 35(4):1276–88. 10.1159/000373950 25721573

[42] Huang J Jiao J Xu W Zhao H Zhang C Shi Y. MiR-155 is Upregulated in Patients With Active Tuberculosis and Inhibits Apoptosis of Monocytes by Targeting FOXO3. Mol Med Rep (2015) 12(5):7102–8. 10.3892/mmr.2015.4250 26324048

[43] Sun Q Shen X Wang P Ma J Sha W. Targeting Cyclophilin-D by miR-1281 Protects Human Macrophages From Mycobacterium Tuberculosis-Induced Programmed Necrosis and Apoptosis. Aging (Albany NY) (2019) 11(24):12661–73. 10.18632/aging.102593 PMC6949086 31884421

[44] Zhang D Yi Z Fu Y. Downregulation of miR-20b-5p Facilitates Mycobacterium Tuberculosis Survival in RAW 264.7 Macrophages Via Attenuating the Cell Apoptosis by Mcl-1 Upregulation. J Cell Biochem (2019) 120(4):5889–96. 10.1002/jcb.27874 30378171

[45] Fu B Xue W Zhang H Zhang R Feldman K Zhao Q. MicroRNA-325-3p Facilitates Immune Escape of Mycobacterium Tuberculosis Through Targeting LNX1 Via NEK6 Accumulation to Promote Anti-Apoptotic STAT3 Signaling. mBio (2020) 11(3). 10.1128/mBio.00557-20 PMC7267881 32487755

[46] Bettencourt P Marion S Pires D Santos LF Lastrucci C Carmo N. Actin-Binding Protein Regulation by microRNAs as a Novel Microbial Strategy to Modulate Phagocytosis by Host Cells: The Case of N-Wasp and miR-142-3p. Front Cell Infect Microbiol (2013) 3:19. 10.3389/fcimb.2013.00019 23760605 PMC3672780

[47] Gutierrez MG Master SS Singh SB Taylor GA Colombo MI Deretic V. Autophagy is a Defense Mechanism Inhibiting BCG and Mycobacterium Tuberculosis Survival in Infected Macrophages. Cell (2004) 119(6):753–66. 10.1016/j.cell.2004.11.038 15607973

[48] Singh SB Davis AS Taylor GA Deretic V. Human IRGM Induces Autophagy to Eliminate Intracellular Mycobacteria. Science (2006) 313(5792):1438–41. 10.1126/science.1129577 16888103

[49] Kumar R Sahu SK Kumar M Jana K Gupta P Gupta UD. MicroRNA 17-5p Regulates Autophagy in Mycobacterium Tuberculosis-Infected Macrophages by Targeting Mcl-1 and STAT3. Cell Microbiol (2016) 18(5):679–91. 10.1111/cmi.12540 26513648

[50] Duan X Zhang T Ding S Wei J Su C Liu H. MicroRNA-17-5p Modulates Bacille Calmette-Guerin Growth in RAW264.7 Cells by Targeting ULK1. PloS One (2015) 10(9):e0138011. 10.1371/journal.pone.0138011 26384021 PMC4575043

[51] Ouimet M Koster S Sakowski E Ramkhelawon B van Solingen C Oldebeken S. Mycobacterium Tuberculosis Induces the miR-33 Locus to Reprogram Autophagy and Host Lipid Metabolism. Nat Immunol (2016) 17(6):677–86. 10.1038/ni.3434 PMC4873392 27089382

[52] Kim JK Yuk JM Kim SY Kim TS Jin HS Yang CS. MicroRNA-125a Inhibits Autophagy Activation and Antimicrobial Responses During Mycobacterial Infection. J Immunol (2015) 194(11):5355–65. 10.4049/jimmunol.1402557 25917095

[53] Chen Z Wang T Liu Z Zhang G Wang J Feng S. Inhibition of Autophagy by MiR-30A Induced by Mycobacteria Tuberculosis as a Possible Mechanism of Immune Escape in Human Macrophages. Jpn J Infect Dis (2015) 68(5):420–4. 10.7883/yoken.JJID.2014.466 25866116

[54] Kim JK Lee HM Park KS Shin DM Kim TS Kim YS. MIR144* Inhibits Antimicrobial Responses Against Mycobacterium Tuberculosis in Human Monocytes and Macrophages by Targeting the Autophagy Protein DRAM2. Autophagy (2017) 13(2):423–41. 10.1080/15548627.2016.1241922 PMC5324854 27764573

[55] Chen DY Chen YM Lin CF Lo CM Liu HJ Liao TL. MicroRNA-889 Inhibits Autophagy To Maintain Mycobacterial Survival in Patients With Latent Tuberculosis Infection by Targeting TWEAK. mBio (2020) 11(1). 10.1128/mBio.03045-19 PMC6989109 31992621

[56] Qu Y Ding S Ma Z Jiang D Xu X Zhang Y. MiR-129-3p Favors Intracellular BCG Survival in RAW264.7 Cells by Inhibiting Autophagy Via Atg4b. Cell Immunol (2019) 337:22–32. 10.1016/j.cellimm.2019.01.004 30782398

[57] Liu F Chen J Wang P Li H Zhou Y Liu H. MicroRNA-27a Controls the Intracellular Survival of Mycobacterium Tuberculosis by Regulating Calcium-Associated Autophagy. Nat Commun (2018) 9(1):4295. 10.1038/s41467-018-06836-4 30327467 PMC6191460

[58] Gu X Gao Y Mu DG Fu EQ. MiR-23a-5p Modulates Mycobacterial Survival and Autophagy During Mycobacterium Tuberculosis Infection Through TLR2/MyD88/NF-κB Pathway by Targeting TLR2. Exp Cell Res (2017) 354(2):71–7. 10.1016/j.yexcr.2017.03.039 28327409

[59] Wang J Yang K Zhou L Minhaowu Wu Y Zhu M. MicroRNA-155 Promotes Autophagy to Eliminate Intracellular Mycobacteria by Targeting Rheb. PloS Pathog (2013) 9(10):e1003697. 10.1371/journal.ppat.1003697 24130493 PMC3795043

[60] Ghorpade DS Leyland R Kurowska-Stolarska M Patil SA Balaji KN. MicroRNA-155 is Required for Mycobacterium Bovis BCG-mediated Apoptosis of Macrophages. Mol Cell Biol (2012) 32(12):2239–53. 10.1128/mcb.06597-11 PMC3372268 22473996

[61] Etna MP Sinigaglia A Grassi A Giacomini E Romagnoli A Pardini M. Mycobacterium Tuberculosis-Induced miR-155 Subverts Autophagy by Targeting ATG3 in Human Dendritic Cells. PloS Pathog (2018) 14(1):e1006790. 10.1371/journal.ppat.1006790 29300789 PMC5771628

[62] Iwai H Funatogawa K Matsumura K Kato-Miyazawa M Kirikae F Kiga K. MicroRNA-155 Knockout Mice are Susceptible to Mycobacterium Tuberculosis Infection. Tuberculosis (Edinb) (2015) 95(3):246–50. 10.1016/j.tube.2015.03.006 25846955

[63] Capel B Swain A Nicolis S Hacker A Walter M Koopman P. Circular Transcripts of the Testis-Determining Gene Sry in Adult Mouse Testis. Cell (1993) 73(5):1019–30. 10.1016/0092-8674(93)90279-y 7684656

[64] Ye CY Chen L Liu C Zhu QH Fan L. Widespread Noncoding Circular RNAs in Plants. New Phytol (2015) 208(1):88–95. 10.1111/nph.13585 26204923

[65] Rybak-Wolf A Stottmeister C Glažar P Jens M Pino N Giusti S. Circular RNAs in the Mammalian Brain Are Highly Abundant, Conserved, and Dynamically Expressed. Mol Cell (2015) 58(5):870–85. 10.1016/j.molcel.2015.03.027 25921068

[66] Kristensen LS Andersen MS Stagsted LVW Ebbesen KK Hansen TB Kjems J. The Biogenesis, Biology and Characterization of Circular RNAs. Nat Rev Genet (2019) 20(11):675–91. 10.1038/s41576-019-0158-7 31395983

[67] Hansen TB Jensen TI Clausen BH Bramsen JB Finsen B Damgaard CK. Natural RNA Circles Function as Efficient microRNA Sponges. Nature (2013) 495(7441):384–8. 10.1038/nature11993 23446346

[68] Conn SJ Pillman KA Toubia J Conn VM Salmanidis M Phillips CA. The RNA Binding Protein Quaking Regulates Formation of CircRNAs. Cell (2015) 160(6):1125–34. 10.1016/j.cell.2015.02.014 25768908

[69] Li Z Huang C Bao C Chen L Lin M Wang X. Exon-Intron Circular RNAs Regulate Transcription in the Nucleus. Nat Struct Mol Biol (2015) 22(3):256–64. 10.1038/nsmb.2959 25664725

[70] Du WW Yang W Liu E Yang Z Dhaliwal P Yang BB. Foxo3 Circular RNA Retards Cell Cycle Progression Via Forming Ternary Complexes With p21 and CDK2. Nucleic Acids Res (2016) 44(6):2846–58. 10.1093/nar/gkw027 PMC4824104 26861625

[71] Vea A Llorente-Cortes V de Gonzalo-Calvo D. Circular RNAs in Blood. Adv Exp Med Biol (2018) 1087:119–30. 10.1007/978-981-13-1426-1_10 30259362

[72] Jafari Ghods F. Circular RNA in Saliva. Adv Exp Med Biol (2018) 1087:131–9. 10.1007/978-981-13-1426-1_11 30259363

[73] Li HM Ma XL Li HG. Intriguing Circles: Conflicts and Controversies in Circular RNA Research. Wiley Interdiscip Rev RNA (2019) 10(5):e1538. 10.1002/wrna.1538 31034768

[74] Haddad G Lorenzen JM. Biogenesis and Function of Circular RNAs in Health and in Disease. Front Pharmacol (2019) 10:428. 10.3389/fphar.2019.00428 31080413 PMC6497739

[75] Wang ZY Guo ZD Li JM Zhao ZZ Fu YY Zhang CM. Genome-Wide Search for Competing Endogenous RNAs Responsible for the Effects Induced by Ebola Virus Replication and Transcription Using a trVLP System. Front Cell Infect Microbiol (2017) 7:479. 10.3389/fcimb.2017.00479 29209594 PMC5702449

[76] Li X Liu CX Xue W Zhang Y Jiang S Yin QF. Coordinated CircRNA Biogenesis and Function With NF90/NF110 in Viral Infection. Mol Cell (2017) 67(2):214–27.e217. 10.1016/j.molcel.2017.05.023 28625552

[77] Liu H Lu G Wang W Jiang X Gu S Wang J. A Panel of CircRNAs in the Serum Serves as Biomarkers for Mycobacterium Tuberculosis Infection. Front Microbiol (2020) 11:1215. 10.3389/fmicb.2020.01215 32582119 PMC7296121

[78] Cui JY Liang HW Pan XL Li D Jiao N Liu YH. Characterization of a Novel Panel of Plasma microRNAs That Discriminates Between Mycobacterium Tuberculosis Infection and Healthy Individuals. PloS One (2017) 12(9):e0184113. 10.1371/journal.pone.0184113 28910318 PMC5598944

[79] Shi Q Wang J Yang Z Liu Y. CircAGFG1modulates Autophagy and Apoptosis of Macrophages Infected by Mycobacterium Tuberculosis Via the Notch Signaling Pathway. Ann Transl Med (2020) 8(10):645. 10.21037/atm.2020-20-3048 32566582 PMC7290638

[80] Huang Z Yao F Liu J Xu J Guo Y Su R. Up-Regulation of circRNA-0003528 Promotes Mycobacterium Tuberculosis Associated Macrophage Polarization Via Down-Regulating miR-224-5p, miR-324-5p and miR-488-5p and Up-Regulating CTLA4. Aging (Albany NY) (2020) 12(24):25658–72. 10.18632/aging.104175 PMC7803570 33318319

[81] Luo HL Pi J Zhang JA Yang EZ Xu H Luo H. Circular RNA TRAPPC6B Inhibits Intracellular Mycobacterium Tuberculosis Growth While Inducing Autophagy in Macrophages by Targeting MicroRNA-874-3p. Clin Transl Immunol (2021) 10(2):e1254. 10.1002/cti2.1254 PMC7890665 33708385

[82] Zhuang ZG Zhang JA Luo HL Liu GB Lu YB Ge NH. The Circular RNA of Peripheral Blood Mononuclear Cells: Hsa_circ_0005836 as a New Diagnostic Biomarker and Therapeutic Target of Active Pulmonary Tuberculosis. Mol Immunol (2017) 90:264–72. 10.1016/j.molimm.2017.08.008 28846924

[83] Huang Z Su R Deng Z Xu J Peng Y Luo Q. Identification of Differentially Expressed Circular RNAs in Human Monocyte Derived Macrophages Response to Mycobacterium Tuberculosis Infection. Sci Rep (2017) 7(1):13673. 10.1038/s41598-017-13885-0 29057952 PMC5651861

[84] Huang ZK Yao FY Xu JQ Deng Z Su RG Peng YP. Microarray Expression Profile of Circular Rnas in Peripheral Blood Mononuclear Cells From Active Tuberculosis Patients. Cell Physiol Biochem (2018) 45(3):1230–40. 10.1159/000487454 29448254

[85] Fu Y Wang J Qiao J Yi Z. Signature of Circular RNAs in Peripheral Blood Mononuclear Cells From Patients With Active Tuberculosis. J Cell Mol Med (2019) 23(3):1917–25. 10.1111/jcmm.14093 PMC6378186 30565391

[86] Yi XH Zhang B Fu YR Yi ZJ. STAT1 and its Related Molecules as Potential Biomarkers in Mycobacterium Tuberculosis Infection. J Cell Mol Med (2020) 24(5):2866–78. 10.1111/jcmm.14856 PMC7077527 32048448

[87] Yi Z Gao K Li R Fu Y. Dysregulated circRNAs in Plasma From Active Tuberculosis Patients. J Cell Mol Med (2018) 22(9):4076–84. 10.1111/jcmm.13684 PMC6111852 29961269

[88] Wagh V Urhekar A Modi D. Levels of microRNA miR-16 and miR-155 are Altered in Serum of Patients With Tuberculosis and Associate With Responses to Therapy. Tuberculosis (Edinb) (2017) 102:24–30. 10.1016/j.tube.2016.10.007 28061948

[89] Ojha R Nandani R Chatterjee N Prajapati VK. Emerging Role of Circular RNAs as Potential Biomarkers for the Diagnosis of Human Diseases. Adv Exp Med Biol (2018) 1087:141–57. 10.1007/978-981-13-1426-1_12 30259364

[90] Zhang L Huang C Guo Y Gou X Hinsdale M Lloyd P. MicroRNA-26b Modulates the NF-κB Pathway in Alveolar Macrophages by Regulating PTEN. J Immunol (2015) 195(11):5404–14. 10.4049/jimmunol.1402933 PMC4655123 26503952

[91] Guttman M Amit I Garber M French C Lin MF Feldser D. Chromatin Signature Reveals Over a Thousand Highly Conserved Large Non-Coding RNAs in Mammals. Nature (2009) 458(7235):223–7. 10.1038/nature07672 PMC2754849 19182780

[92] Guttman M Russell P Ingolia NT Weissman JS Lander ES. Ribosome Profiling Provides Evidence That Large Noncoding RNAs do Not Encode Proteins. Cell (2013) 154(1):240–51. 10.1016/j.cell.2013.06.009 PMC3756563 23810193

[93] Ulitsky I Bartel DP. lincRNAs: Genomics, Evolution, and Mechanisms. Cell (2013) 154(1):26–46. 10.1016/j.cell.2013.06.020 23827673 PMC3924787

[94] Chen J Shishkin AA Zhu X Kadri S Maza I Guttman M. Evolutionary Analysis Across Mammals Reveals Distinct Classes of Long Non-Coding RNAs. Genome Biol (2016) 17:19. 10.1186/s13059-016-0880-9 26838501 PMC4739325

[95] Engreitz JM Ollikainen N Guttman M. Long Non-Coding RNAs: Spatial Amplifiers That Control Nuclear Structure and Gene Expression. Nat Rev Mol Cell Biol (2016) 17(12):756–70. 10.1038/nrm.2016.126 27780979

[96] Ransohoff JD Wei Y Khavari PA. The Functions and Unique Features of Long Intergenic Non-Coding RNA. Nat Rev Mol Cell Biol (2018) 19(3):143–57. 10.1038/nrm.2017.104 PMC5889127 29138516

[97] Iyer MK Niknafs YS Malik R Singhal U Sahu A Hosono Y. The Landscape of Long Noncoding RNAs in the Human Transcriptome. Nat Genet (2015) 47(3):199–208. 10.1038/ng.3192 25599403 PMC4417758

[98] McDonel P Guttman M. Approaches for Understanding the Mechanisms of Long Noncoding RNA Regulation of Gene Expression. Cold Spring Harb Perspect Biol (2019) 11(12). 10.1101/cshperspect.a032151 PMC6886450 31791999

[99] Atianand MK Hu W Satpathy AT Shen Y Ricci EP Alvarez-Dominguez JR. A Long Noncoding RNA LincRNA-EPS Acts as a Transcriptional Brake to Restrain Inflammation. Cell (2016) 165(7):1672–85. 10.1016/j.cell.2016.05.075 PMC5289747 27315481

[100] Carpenter S Aiello D Atianand MK Ricci EP Gandhi P Hall LL. A Long Noncoding RNA Mediates Both Activation and Repression of Immune Response Genes. Science (2013) 341(6147):789–92. 10.1126/science.1240925 PMC4376668 23907535

[101] Subuddhi A Kumar M Majumder D Sarkar A Ghosh Z Vasudevan M. Unraveling the Role of H3K4 Trimethylation and LncRNA HOTAIR in SATB1 and DUSP4-dependent Survival of Virulent Mycobacterium Tuberculosis in Macrophages. Tuberculosis (Edinb) (2020) 120:101897. 10.1016/j.tube.2019.101897 32090865

[102] Yan H Xu R Zhang X Wang Q Pang J Zhang X. Identifying Differentially Expressed Long Non-Coding RNAs in PBMCs in Response to the Infection of Multidrug-Resistant Tuberculosis. Infect Drug Resist (2018) 11:945–59. 10.2147/idr.S154255 PMC6047615 30034244

[103] Pawar K Hanisch C Palma Vera SE Einspanier R Sharbati S. Down Regulated LncRNA MEG3 Eliminates Mycobacteria in Macrophages Via Autophagy. Sci Rep (2016) 6:19416. 10.1038/srep19416 26757825 PMC4725832

[104] Ke Z Lu J Zhu J Yang Z Jin Z Yuan L. Down-Regulation of lincRNA-EPS Regulates Apoptosis and Autophagy in BCG-infected RAW264.7 Macrophages Via JNK/MAPK Signaling Pathway. Infect Genet Evol (2020) 77:104077. 10.1016/j.meegid.2019.104077 31669366

[105] Sun W Lou H Cao J Wang P Sha W Sun Q. LncRNA MEG3 Control Mycobacterium Tuberculosis Infection Via Controlled MiR-145-5p Expression and Modulation of Macrophages Proliferation. Microb Pathog (2020) 149:104550. 10.1016/j.micpath.2020.104550 33035634

[106] Li M Cui J Niu W Huang J Feng T Sun B. Long Non-Coding PCED1B-AS1 Regulates Macrophage Apoptosis and Autophagy by Sponging miR-155 in Active Tuberculosis. Biochem Biophys Res Commun (2019) 509(3):803–9. 10.1016/j.bbrc.2019.01.005 30621915

[107] Bai H Wu Q Hu X Wu T Song J Liu T. Clinical Significance of lnc-AC145676.2.1-6 and lnc-TGS1-1 and Their Variants in Western Chinese Tuberculosis Patients. Int J Infect Dis (2019) 84:8–14. 10.1016/j.ijid.2019.04.018 31028876

[108] Sun Q Shen X Ma J Lou H Sha W. LncRNA NEAT1 Participates in Inflammatory Response in Macrophages Infected by Mycobacterium Tuberculosis Through Targeted Regulation of miR-377-3p. Microb Pathog (2021) 150:104674. 10.1016/j.micpath.2020.104674 33271233

[109] Wang Y Zhong H Xie X Chen CY Huang D Shen L. Long Noncoding RNA Derived From CD244 Signaling Epigenetically Controls CD8+ T-Cell Immune Responses in Tuberculosis Infection. Proc Natl Acad Sci U S A (2015) 112(29):E3883–92. 10.1073/pnas.1501662112 PMC4517270 26150504

[110] Munschauer M Nguyen CT Sirokman K Hartigan CR Hogstrom L Engreitz JM. The NORAD lncRNA Assembles a Topoisomerase Complex Critical for Genome Stability. Nature (2018) 561(7721):132–6. 10.1038/s41586-018-0453-z 30150775

[111] Tay Y Rinn J Pandolfi PP. The Multilayered Complexity of ceRNA Crosstalk and Competition. Nature (2014) 505(7483):344–52. 10.1038/nature12986 PMC4113481 24429633

[112] Salmena L Poliseno L Tay Y Kats L Pandolfi PP. A ceRNA Hypothesis: The Rosetta Stone of a Hidden RNA Language? Cell (2011) 146(3):353–8. 10.1016/j.cell.2011.07.014 PMC3235919 21802130

[113] Chen ZL Wei LL Shi LY Li M Jiang TT Chen J. Screening and Identification of lncRNAs as Potential Biomarkers for Pulmonary Tuberculosis. Sci Rep (2017) 7(1):16751. 10.1038/s41598-017-17146-y 29196714 PMC5711916

[114] Zhang X Guo J Fan S Li Y Wei L Yang X. Screening and Identification of Six Serum microRNAs as Novel Potential Combination Biomarkers for Pulmonary Tuberculosis Diagnosis. PloS One (2013) 8(12):e81076. 10.1371/journal.pone.0081076 24349033 PMC3857778

[115] Jasenosky LD Scriba TJ Hanekom WA Goldfeld AE. T Cells and Adaptive Immunity to Mycobacterium Tuberculosis in Humans. Immunol Rev (2015) 264(1):74–87. 10.1111/imr.12274 25703553

[116] Lin PL Flynn JL. CD8 T Cells and Mycobacterium Tuberculosis Infection. Semin Immunopathol (2015) 37(3):239–49. 10.1007/s00281-015-0490-8 PMC4439333 25917388

[117] Fu Y Gao K Tao E Li R Yi Z. Aberrantly Expressed Long Non-Coding Rnas In CD8(+) T Cells Response to Active Tuberculosis. J Cell Biochem (2017) 118(12):4275–84. 10.1002/jcb.26078 28422321

[118] Fu Y Xu X Xue J Duan W Yi Z. Deregulated lncRNAs in B Cells From Patients With Active Tuberculosis. PloS One (2017) 12(1):e0170712. 10.1371/journal.pone.0170712 28125665 PMC5268381

